# Integrate multi-omics data with biological interaction networks using Multi-view Factorization AutoEncoder (MAE)

**DOI:** 10.1186/s12864-019-6285-x

**Published:** 2019-12-20

**Authors:** Tianle Ma, Aidong Zhang

**Affiliations:** 1 Department of Computer Science and Engineering, University at Buffalo, 338 Davis Hall, Buffalo, 14260 NY USA; 2 Department of Computer Science, University of Virginia, 509 Rice Hall, Charlottesville, 22904 VA USA

**Keywords:** Multi-omics data, Biological interaction networks, Deep learning, Multi-view learning, Autoencoder, Data integration, Graph regularization

## Abstract

**Background:**

Comprehensive molecular profiling of various cancers and other diseases has generated vast amounts of multi-omics data. Each type of -omics data corresponds to one feature space, such as gene expression, miRNA expression, DNA methylation, etc. Integrating multi-omics data can link different layers of molecular feature spaces and is crucial to elucidate molecular pathways underlying various diseases. Machine learning approaches to mining multi-omics data hold great promises in uncovering intricate relationships among molecular features. However, due to the “big p, small n” problem (i.e., small sample sizes with high-dimensional features), training a large-scale generalizable deep learning model with multi-omics data alone is very challenging.

**Results:**

We developed a method called Multi-view Factorization AutoEncoder (MAE) with network constraints that can seamlessly integrate multi-omics data and domain knowledge such as molecular interaction networks. Our method learns feature and patient embeddings simultaneously with deep representation learning. Both feature representations and patient representations are subject to certain constraints specified as regularization terms in the training objective. By incorporating domain knowledge into the training objective, we implicitly introduced a good inductive bias into the machine learning model, which helps improve model generalizability. We performed extensive experiments on the TCGA datasets and demonstrated the power of integrating multi-omics data and biological interaction networks using our proposed method for predicting target clinical variables.

**Conclusions:**

To alleviate the overfitting problem in deep learning on multi-omics data with the “big p, small n” problem, it is helpful to incorporate biological domain knowledge into the model as inductive biases. It is very promising to design machine learning models that facilitate the seamless integration of large-scale multi-omics data and biomedical domain knowledge for uncovering intricate relationships among molecular features and clinical features.

## Background

With the fast adoption of Next Generation Sequencing (NGS) technologies, petabytes of genomic, transcriptomic, proteomic, and epigenomic data (collectively called multi-omics data) have been accumulated in the past decade. Notably, The Cancer Genome Atlas (TCGA) Network [1] alone had generated over one petabyte of multi-omics data for comprehensive molecular profiling of over 11,000 patients from 33 cancer types. Multi-omics data includes multiple types of -omics data, each of which represents one view and has a different feature set (for instance, gene expressions, miRNA expressions, and so on). Since multiple views for the same patients can provide complementary information, integrative analysis of multi-omics data with machine learning approaches has great potentials to elucidate the molecular underpinning of disease etiology. However, due to the “big p, small n” problem, many statistical machine learning approaches that require lots of training data may fail to extract true signals from multi-omics data alone.

Deep learning has achieved great success in computer vision, speech recognition, natural language processing and many other fields in the past decade [2]. However, deep learning models often require large amounts of annotated training data with clearly defined structures (such as images, audio, and natural languages), and cannot be directly applied to multi-omic data with unclear structures among features and a small sample size. Novel model architectures and learning strategies need to be invented to address the challenge of learning from multi-omics data with heterogeneous features and the “big p, small n” problem.

In this paper, we present a framework called Multi-view Factorization AutoEncoder (MAE) with network constraints [3], combining multi-view learning [4] and matrix factorization [5] with deep learning for integrating multi-omics data with biological domain knowledge. The MAE model consists of multiple autoencoders as submodules (one for each data view), and a submodule that combines individual views. The model facilitates learning feature and patient embeddings simultaneously with deep representation learning. Importantly, we incorporate domain knowledge such as biological interaction networks into the model training objective to ensure the learned feature embeddings are consistent with the domain knowledge.

Besides the molecular interaction networks, we can construct multiple patient similarity networks based on the learned patient embeddings from individual views. We included patient similarity network constraints to ensure these similarity networks for the same set of patients are consistent with each other. Equipped with feature interaction and patient similarity network constraints, our model achieved better performance than traditional machine learning methods and conventional deep learning models without using domain knowledge on the TCGA datasets [1].

### Related work

Many genetic disease studies focus on molecular characterization of individual disease types [1, 6], employing mainly statistical analyses to find associations among molecular and clinical features. Machine learning has been applied to individual -omics data types [7] and to integrate multi-omics data [8, 9]. Because most existing deep learning models cannot handle the “big p, small n” problem effectively, many traditional machine learning methods (such as logistic regression [7], random forest [8], and similarity network fusion [9]) have been applied to -omics data.

Comprehensive multi-omics data analysis with machine learning has been a frontier in cancer genomics [1, 10, 11]. Unsupervised clustering approaches (such as iCluster [12], SNF [13], ANF [14], etc.) are popular for multi-omics data analysis as annotated labels are often lacking in biomedical data. Probabilistic models [12] and network-based regularization [15] have been employed to learn from multi-omics data. Recently, deep learning has been applied to sequencing data [16, 17], imaging data [18], medical records [19], etc. However, most existing deep learning methods focused on individual data types instead of integrating multi-omics data. Multi-view learning provides a natural framework for learning from multimodal data. Typical techniques for multi-view learning include co-training, co-regularization, and margin consistency approaches [4]. Combining deep learning with multi-view learning more effectively is still active research [4]. There are multiple ways to incorporate biological networks as inductive biases into a deep learning model. Besides network regularization approaches, directly encoding biological networks into the model architecture is also possible [20, 21], which usually requires subcellular hierarchical molecular networks as the prior knowledge. Because high-quality human data is lacking (human biological interaction networks such as protein-protein interaction networks are still incomplete and noisy), network regularization approaches are often preferable to directly encoding the noisy interaction network into the model architecture.

Multi-modality deep learning [22] has been successfully applied to integrate audio and video features [23] by employing shared feature representations. However, many multi-modality deep learning models still rely on large amounts of training data and do not facilitate knowledge integration. Our method can learn feature and patient embeddings simultaneously with the integration of domain knowledge to learn robust and generalizable deep learning models.

Many multi-view learning techniques have been proposed [24, 25]. Many of these methods learn transformations that map each view to a latent space and reconstruct the original data from the latent space representation (i.e., adopting an AutoEncoder architecture). Importantly, they may add additional constraints to ensure the latent features for multiple views are highly correlated [24]. Our model also adopted the Multi-view AutoEncoder architecture as the model backbone, but we chose different regularization schemes for incorporating domain knowledge as inductive biases into the model. We do not assume the latent spaces learned for each view to be “canonically correlated”. Instead, the learned feature representations should be consistent with the domain knowledge such as gene-gene and miRNA-miRNA interaction networks. As the gene-gene interaction network and the miRNA-miRNA interaction network are very different, the corresponding gene and miRNA feature interactions can be very different as well. Importantly, we are focusing on the multi-omics data, of which each feature (such as a gene) has a clear biological meaning and the feature interactions have been captured as domain knowledge, while many other proposed multi-view learning methods deal with data without “biologically meaningful” features (for example, in image data, individual pixels are not informative at all, but their arrangement and structure do contain information). While other widely used multi-view learning methods [22, 24, 25] focus on how to effectively utilize feature correlation among different views to improve model performance, our main focus in this paper is to demonstrate that biological interaction networks as an "external" domain knowledge source can be effectively incorporated into deep learning models through network regularization to improve model generalizability for multi-omics data analysis.

Our main contribution can be summarized as follows. We proposed a Multi-view AutoEncoder model with network constraints for the integrative analysis of multi-omics data. Our model learns good representations for both molecular entities and patients simultaneously and facilitates mining relationships among molecular features and clinical features. Most importantly, we demonstrated that “external” domain knowledge sources such as biological interaction networks can be incorporated into the model as inductive biases, which could improve model generalizability and reduce the risk of overfitting in the “big p, small n” problem. We devised novel network regularizers that will “force” the learned feature representations to be consistent with domain knowledge, effectively reducing the search space for good feature embeddings. We have performed extensive experiments and showed that the models trained with domain knowledge outperformed those without using domain knowledge. Our work provides a proof-of-concept framework for unifying data-driven and knowledge-driven approaches for mining multi-omics data with biological knowledge.

## Methods and implementation

Our method builds upon matrix factorization [5], multi-view learning, and deep learning. We will describe each component in the following section.

**Some notations**Given *N* samples and *V* types of -omics data, we can often represent the data using *V* sample-feature matrices: $\mathbf {M}^{(i)} \in \mathbb {R}^{N \times p^{(i)}}, i=1,2,\cdots, V$. Each matrix corresponds to one data view, and *p*^(*i*)^ is the feature dimension for view *i*.

Before describing Multi-view Factorization AutoEncoder, we first discuss how to process individual views. For ease of description, we drop the superscript ^(·)^ when dealing with a single view. For matrix **M**,*M*_*ij*_ represents the element of *i*th row and *j*th column, **M**_*i*,·_ represents the *i*th row vector, and **M**_·,*j*_ represents *j*th column vector.

Let $\mathbf {M} \in \mathbb {R}^{N \times p}$ be a feature matrix, with each row corresponding to a sample and each column corresponding to a feature. The features are often not independent. We represent the interactions among these features with a network $\mathbf {G} \in \mathbb {R}^{p \times p}$. For instance, if these features correspond to protein expressions, then **G** will be a protein-protein interaction network, which is available in public databases such as STRING [26] and Reactome [27]. **G** can be an unweighted graph or a weighted graph with non-negative elements. Let **D** be a diagonal matrix with $D_{ii}=\sum _{j=1}^{p} G_{ij}$, then the graph Laplacian of **G** is **L**_*G*_=**D**−**G**.

### Low-rank matrix factorization

Matrix factorization techniques [5] are widely used for clustering and dimensionality reduction. In many real-world applications, **M** often has a low rank. As a result, low-rank matrix factorization can be used for dimensionality reduction and clustering:

**M**≈**XY**, where $\mathbf {X} \in \mathbb {R}^{N \times k}, \mathbf {Y} \in \mathbb {R}^{k \times p}, k< p$

Some additional constraints are often added as regularizers in the objective function or enforced in the learning algorithm to find a good solution {**X**,**Y**}. For instance, when **M** is non-negative, Non-negative Matrix Factorization (NMF) [28] is often a “natural” choice to ensure both **X** and **Y** are non-negative.

Generally speaking, the objective function can be formulated as follows:

1 $$ \arg \min_{\mathbf{X}, \mathbf{Y}} \left\Vert \mathbf{M} - \mathbf{X} \mathbf{Y} \right\Vert^{2}_{F} + \lambda R(\mathbf{X}, \mathbf{Y})  $$

In Eq. 1, *R*(**X**,**Y**) is a regularization term for **X** and **Y**. For instance, *R*(**X**,**Y**) can include *L*_1_ and *L*_2_ norms for **X** and **Y**. In addition, structural constraints based on biological interaction networks can also be incorporated into *R*(**X**,**Y**).

**Interpretation**$\mathbf {X} \in \mathbb {R}^{N \times k}$ can be regarded as a sample-factor matrix and the inherent non-redundant representation of *N* samples, with each column corresponding to an independent factor. These *k* factors are often latent variables. $\mathbf {Y} \in \mathbb {R}^{k \times p}$ can be seen as a linear transformation matrix. The *k* rows of **Y** can be regarded as a basis for the underlying factor space. The observable feature matrix **M** is generated by a linear transformation **Y** from **X**. In a sense, this formulation can be seen as a shallow linear generative model.

**Limitations**The limitations of matrix factorization techniques often stem from their “shallow” linear structure with a limited representation power. In many real-world applications, however, we need to learn a complex nonlinear transformation. Deep neural networks are often good at approximating any complex nonlinear transformations with appropriate training on a sufficiently large dataset.

### Non-linear factorization with AutoEncoder

As simple matrix factorization techniques are limited to model complex nonlinear relationships, we can use an Autoencoder to reconstruct the observable sample-feature matrix **M**, as it can approximate more complex nonlinear transformations well.

The entire Autoencoder is a multi-layer neural network with a encoder and a decoder. We use a neural network with parameter *Θ*_*e*_ as the encoder:

2 $$ Encoder(\mathbf{M}, \mathbf{\Theta_{e}}) = \mathbf{X} \in \mathbb{R}^{N \times k}  $$

Here **X** can be regarded as a factor matrix containing the essential information for all *N* samples. The encoder network will transform the observable sample-feature matrix **M** to its latent representation **X**. The decoder reconstructs the original data from the latent representation.

3 $$  Decoder(\mathbf{X}, \mathbf{\Theta_{d}}) = \mathbf{Z} \in \mathbb{R}^{N \times p}  $$

In our framework, for the convenience of incorporating biological interaction networks into the framework, the encoder (Eq. 2) contains all layers but the last one, and the decoder is the last linear layer. The parameter of decoder (Eq. 3) is a linear transformation matrix same as in matrix factorization:

4 $$  \mathbf{\Theta_{d}} = \mathbf{Y} \in \mathbb{R}^{k \times p}  $$

The input sample-feature matrix can be reconstructed as

5 $$  \mathbf{Z} = Encoder(\mathbf{M}, \mathbf{\Theta_{e}}) \cdot \mathbf{Y} = \mathbf{X} \mathbf{Y}  $$

The reconstruction error can be computed as: $\left \Vert \mathbf {M} - \mathbf {Z} \right \Vert ^{2}_{F}$.

Different from matrix factorization–which can be regarded as a one-layer AutoEncoder, the encoder in our framework is a multi-layer neural network that can learn complex nonlinear transformations through backpropagation. Moreover, the encoder output **X** can be regarded as the learned patient representations for *N* samples, and **Y** can be seen as the learned feature representations. With the learned patient and feature representations, we can calculate patient similarity networks and feature interaction networks, and add network regularizers to the objective function.

### Incorporate biological knowledge as network regularizers

We aim to incorporate biological knowledge such as molecular interaction networks into our model as inductive biases to increase model generalizability. Denote $\mathbf {G} \in \mathbb {R}^{p \times p}$ as the interaction matrix among *p* genomic features, which can be obtained from biological databases such as STRING [26] and Reactome [27].

Since our model can learn a feature representation **Y**, this representation should ideally be “consistent” with the biological interaction network corresponding to these features. We use a graph Laplacian regularizer to minimize the inconsistency between the learned feature representation **Y** and the feature interaction network **G**:

6 $$  Trace\left(\mathbf{Y} \mathbf{L}_{G} \mathbf{Y}^{T}\right) = \frac{1}{2} \sum_{i=1}^{p} \sum_{j=1}^{p} G_{ij} \lVert \mathbf{Y}_{\cdot, i} - \mathbf{Y}_{\cdot, j} \rVert^{2}  $$

**L**_*G*_ is the graph Laplacian matrix of **G** in Eq. 6. *G*_*ij*_≥0 captures how “similar” feature *i* and feature *j* are. Each feature *i* is represented as a *k*-dimensional vector **Y**_·,*i*_. We can calculate the Euclidean distance between feature *i* and *j* as ∥**Y**_·,*i*_−**Y**_·,*j*_∥. The term *Trace*(**YL**_*G*_**Y**^*T*^) is a surrogate for measuring the inconsistency between the learned feature representation **Y** and the known feature interaction network **G**. When **Y** is highly inconsistent with **G**, the loss term *Trace*(**YL**_*G*_**Y**^*T*^), which accounts for the level of inconsistency between the learned feature representation and the biological interaction network, will be large. Therefore, minimizing the loss function can effectively reduce the inconsistency between the learned feature representation and the biological interaction network.

The objective function incorporating biological interaction networks through the graph Laplacian regularizer is as follows:

7 $$  \arg \min_{\mathbf{\Theta_{e}}, \mathbf{Y}} \left\Vert \mathbf{M} - \mathbf{Z} \right\Vert^{2}_{F} + \alpha \ Trace\left(\mathbf{Y} \cdot \mathbf{L}_{G} \cdot \mathbf{Y}^{T}\right)  $$

In Eq. 7, *α*≥0 is a hyperparameter as the weight for the network regularization term. In practice, we normalize **G** and **Y** so that the *Trace*(**Y**·**L**_*G*_·**Y**^*T*^) is within the range of [0,1]. In the implementation of our model, we set $\lVert \mathbf {G} \rVert _{F} = 1, \lVert \mathbf {Y}_{\cdot, i} \rVert = \frac {1}{\sqrt {p}}, i=1,2,\cdots, p$ (this also means ∥**Y**∥_*F*_=1). This facilitates easy multi-view integration since all the network regularizers from individual views are on the same scale.

#### Measuring feature similarity with mutual information

Eq. 6 uses Euclidean distance to measure the dissimilarity between learned feature representations. Euclidean distance relies on the inner product operator, which is essentially linear. The fact that two molecular entities interact with each other does not imply that they should have very similar feature representations or a small Euclidean distance. Mutual information can be a better metric quantifying if two molecular entities interact with each other.

Let’s briefly review the definition of mutual information between two random variables *X* and *Y*.

$$\begin{array}{*{20}l} I(X, Y) &= H(X) - H(X|Y) \\ &= H(Y) - H(Y|X) \\ &= H(X) + H(Y) - H(X,Y) \end{array} $$

For discrete random variable *X*∼*P*(*x*) (*P*(*x*) is the discrete probability distribution of *X*), the entropy of *X* is defined as $H(X) = \sum _{x} P(x) log\frac {1}{P(x)}$.

The observed sample-feature matrix $\mathbf {M} \in \mathbb {R}^{N \times p}$ can be used to measure the pair-wise mutual information scores between feature *i* and *j*: *MutualInfo*(**M**_·,*i*_,**M**_·,*j*_). However, due to measurement noise and error, this may not be accurate.

Ideally, the reconstructed signal with the proposed autoencoder model should reduce the noise in the data. Thus we can calculate pair-wise normalized mutual information scores using the reconstructed signal **Z** (Eq. 5):

$$\mathbf{K} = PairwiseMutualInformation(\mathbf{Z}) \in \mathbb{R}^{p \times p} $$

**K** can be regarded as a learned similarity matrix based on mutual information. Again we want to ensure that the learned similarity matrix is consistent with the known biological interaction network **G**. We can estimate the consistency between **G** and **K** as:

8 $$  \left \lVert \mathbf{G} \odot \mathbf{K} \right \rVert^{2}_{F}  $$

⊙ is element-wise matrix multiplication. As **G** and **K** are normalized feature interaction network and pairwise feature mutual information matrix, the norm of their element-wise multiplication can be an estimate of the consistency between **G** and **K**. We inject this mutual information regularization term into Eq. 9:

9 $$  \begin{aligned} \underset{\mathbf{\Theta_{e}}, \mathbf{Y}}{\arg \min} &\left\Vert \mathbf{M} - Encoder(\mathbf{M}, \mathbf{\Theta_{e}}) \cdot \mathbf{Y} \right\Vert^{2}_{F} \\ &+ \alpha \ Trace\left(\mathbf{Y} \cdot \mathbf{L}_{G} \cdot \mathbf{Y}^{T}\right) \\ &- \gamma \left \lVert \mathbf{G} \odot \mathbf{K} \right \rVert^{2}_{F} \end{aligned}  $$

*α*,*γ* are non-negative hyperparameters. There are numerical methods to measure the mutual information between two continuous high-dimensional random variables. The simplest approach is to divide the continuous space into small bins and discretize the variables with these bins. In order to estimate mutual information from data accurately, a large sample size is needed. Due to the difficulty in accurately calculating mutual information based on a limited number of data points, we do not include mutual information term in the following discussion and leave this for future work.

### Multi-view factorization AutoEncoder with network constraints

We have given the objective function for a single view in Eq. 7. For multiple views, the objective can be formulated as follows:

10 $$  \begin{aligned} \underset{\{\mathbf{\Theta_{e}}^{(v)}, \mathbf{Y}^{(v)}\}}{\arg \min} & \sum_{v=1}^{V} \left(\left\Vert \mathbf{M}^{(v)} - Encoder\left(\mathbf{M}^{(v)}, \mathbf{\Theta_{e}}^{(v)}\right) \cdot \mathbf{Y}^{(v)} \right\Vert^{2}_{F} \right.\\ &\left. + \alpha \ Trace\left(\mathbf{Y}^{(v)} \cdot \mathbf{L}_{G^{(v)}} \cdot \mathbf{Y}^{(v)^{T}}\right) \right) \end{aligned}  $$

Note we use a separate autoencoder for each view. We try to minimize the reconstruction loss and feature interaction network regularizers for all views in Eq. 10.

Here *Encoder*(**M**^(*v*)^,*Θ*_*e*_^(*v*)^)=**X**^(*v*)^ can be seen as the learned latent representation for *N* samples. We can derive patient similarity network **S**^(*v*)^ (which can also be used for clustering patients into groups) from **X**^(*v*)^. Multiple approaches can be employed to calculate a patient similarity network. For example, we can use cosine similarity:

11 $$ S_{ij}=\frac{\lvert \mathbf{X}_{i, \cdot} \cdot \mathbf{X}_{j, \cdot} \rvert}{\lVert \mathbf{X}_{i, \cdot}\rVert \cdot \lVert \mathbf{X}_{j, \cdot} \rVert}  $$

We can get a patient similarity network **S**^(*v*)^ for each view *v* (Eq. 11 omits the superscript for clarity). Moreover, the outputs of multiple encoders can be “fused” together for supervised learning.

12 $$ \mathbf{X} = \sum_{v=1}^{V} \mathbf{X}^{(v)} = \sum_{v=1}^{V}Encoder\left(\mathbf{M}^{(v)}, \mathbf{\Theta_{e}}^{(v)}\right)  $$

This idea is similar to ResNet [29]. Another approach is to concatenate all views together like DenseNet [30]. We have tried using both in our experiments and the results are not significantly different.

With the fused view **X**, we can again calculate the patient similarity network **S**_*X*_ using Eq. 11. Moreover, since **S**_*X*_, and**S**^(*v*)^,*v*=1,2,⋯,*V* are for the same set of patients, we can fuse them together using affinity network fusion [14]:

13 $$  \mathbf{S} = \frac{1}{V+1} \left(\sum_{i=1}^{V} \mathbf{S}^{(v)} + \mathbf{S}_{X} \right)  $$

Similar to the feature interaction network regularizer (Eq. 6), we also include a regularization term on the patient view similarity:

14 $$  Trace\left(\mathbf{X}^{(v)^{T}} \cdot \mathbf{L}_{S} \cdot \mathbf{X}^{(v)}\right)  $$

Here **L**_*S*_ is the graph Laplacian of **S**. Adding this term to Eq. 10, we get the new objective function:

15 $$  \begin{aligned} \underset{\{\mathbf{\Theta_{e}}^{(v)}, \mathbf{Y}^{(v)}\}}{\arg \min} \sum_{v=1}^{V} &\left(\left\Vert \mathbf{M}^{(v)} - Encoder\left(\mathbf{M}^{(v)}, \mathbf{\Theta_{e}}^{(v)}\right) \cdot \mathbf{Y}^{(v)} \right\Vert^{2}_{F} \right.\\ & + \alpha \ Trace\left(\mathbf{Y}^{(v)} \cdot \mathbf{L}_{G^{(v)}} \cdot \mathbf{Y}^{(v)^{T}}\right)\\ & \left.+ \beta\ Trace\left(\mathbf{X}^{(v)^{T}} \cdot \mathbf{L}_{S} \cdot \mathbf{X}^{(v)}\right)\right) \end{aligned}  $$

For each type of -omic data, there is one corresponding feature interaction network **G**^(*v*)^. Different molecular interaction networks involve distinct feature sets and thus cannot be directly merged. However, patient similarity networks are about the same set of patients, and therefore can be combined to get a fused patient similarity network **S** using techniques such as affinity network fusion [14]. Our framework uses both molecular interaction networks and patient similarity networks for regularized learning.

### Supervised learning with multi-view factorization autoencoder

With multi-view data and feature interaction networks, our framework with the objective function Eq. 7 can be used for unsupervised learning. When labeled data is available, we can use our model for supervised learning by adding another loss term to Eq. 7:

16 $$  \begin{aligned} &\underset{\{\mathbf{\Theta_{e}}^{(v)}, \mathbf{Y}^{(v)}\}}{\arg \min} \mathcal{L}(\mathbf{T}, \left(\sum_{v=1}^{V}Encoder\left(\mathbf{M}^{(v)}, \mathbf{\Theta_{e}}^{(v)}\right)\right) \cdot \mathbf{C})\\ &+ \eta \sum_{v=1}^{V} \left(\left\Vert \mathbf{M}^{(v)} - Encoder\left(\mathbf{M}^{(v)}, \mathbf{\Theta_{e}}^{(v)}\right) \cdot \mathbf{Y}^{(v)} \right\Vert^{2}_{F}\right)\\ & + \alpha \sum_{v=1}^{V} Trace\left(\mathbf{Y}^{(v)} \cdot \mathbf{L}_{G^{(v)}} \cdot \mathbf{Y}^{(v)^{T}}\right) \\ & + \beta\ \sum_{v=1}^{V} Trace\left(\mathbf{X}^{(v)^{T}} \cdot \mathbf{L}_{S} \cdot \mathbf{X}^{(v)}\right) \end{aligned}  $$

The first term $\mathcal {L}(\mathbf {T}, \left (\sum _{v=1}^{V}Encoder\left (\mathbf {M}^{(v)}, \mathbf {\Theta _{e}}^{(v)}\right)\right) \cdot \mathbf {C})$ is the classification loss (e.g., cross entropy loss) or regression loss (e.g., mean squared error for continuous target variables) for supervised learning. **T** is the true class labels or other target variables available for training the model.

As in Eq. 12, $\sum _{v=1}^{V}Encoder\left (\mathbf {M}^{(v)}, \mathbf {\Theta _{e}}^{(v)}\right)$ is the sum of the last hidden layers of *V* autoencoders. This also represents the learned patient representations combining multiple views. **C** is the weights for the last fully connected layer typically used in neural network models for classification tasks. The second term $\sum _{v=1}^{V} \left (\left \Vert \mathbf {M}^{(v)} - Encoder\left (\mathbf {M}^{(v)}, \mathbf {\Theta _{e}}^{(v)}\right) \cdot \mathbf {Y}^{(v)} \right \Vert ^{2}_{F}\right)$ is the reconstruction loss for all the submodule autoencoders. The third and four terms are the graph Laplacian constraints for molecular interaction networks and patient similarity networks as in Eq. 6 and Eq. 14. *η*,*α*,*β* are non-negative hyperparameters adjusting the weights of the reconstruction loss, feature interaction network loss, and patient similarity network loss.

**Fig. 1:**
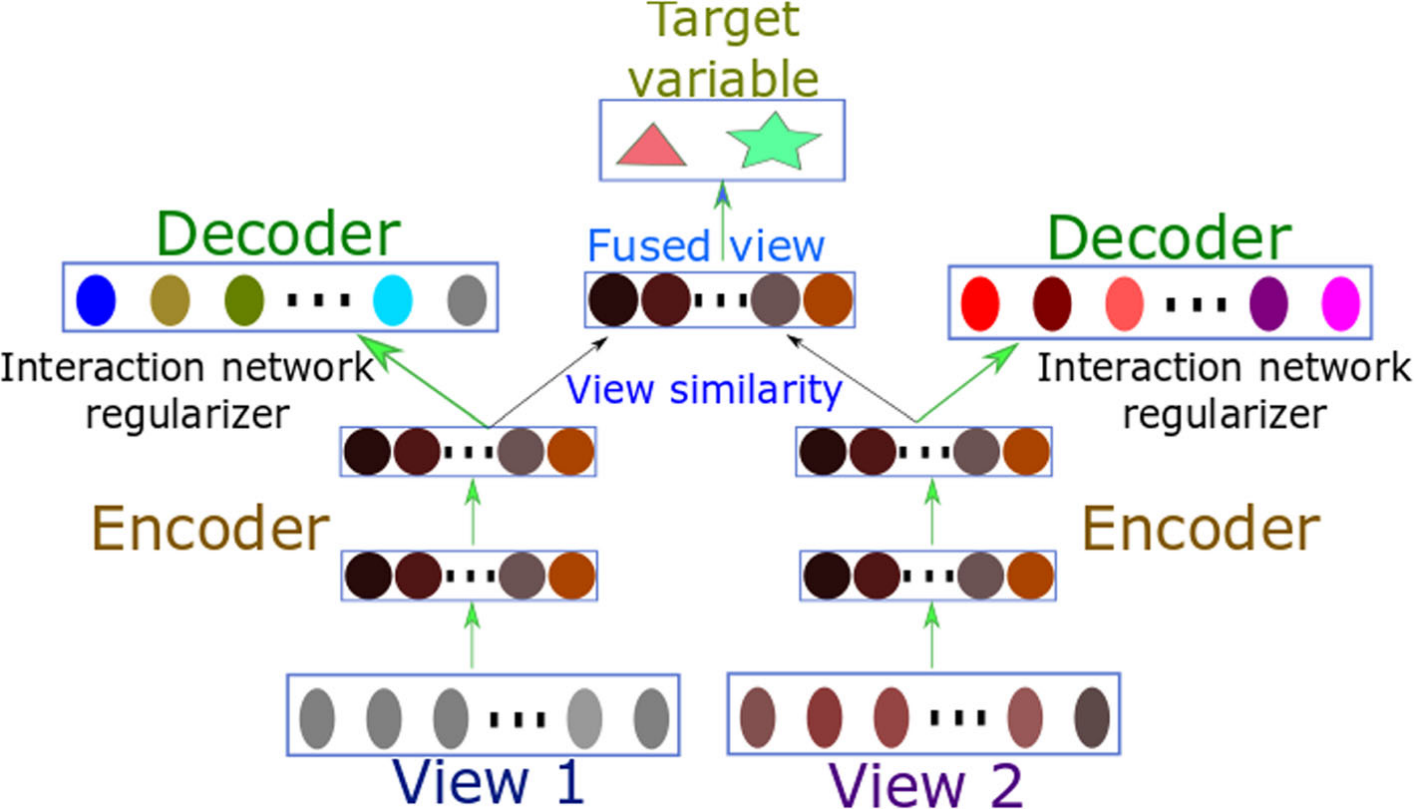
A simple illustration of the proposed framework with two data views, each with an encoder and a decoder. Different views are fused in the latent space and the fused view is used for supervised learning. Feature interaction networks are incorporated as regularizers into the training objective

A simple illustration of the whole framework combining two views with two-hidden-layer autoencoders is depicted in Fig. 1. The whole framework is end-to-end differentiable. We implemented the model using PyTorch (https://github.com/BeautyOfWeb/Multiview-AutoEncoder).

## Results and discussion

### Datasets

We downloaded and processed two datasets from The Cancer Genome Atlas (TCGA): Bladder Urothelial Carcinoma (BLCA) and Brain Lower Grade Glioma (LGG). 338 patients from the BLCA project and 423 patients from the LGG project were selected for downstream analysis, all of which have gene expression, miRNA expression, protein expression, and DNA methylation as well as clinical data available.

#### Target clinical variable

The main target variable is the Progression-Free Interval (PFI) event. PFI is a derived clinical (binary) outcome endpoint [31], which is relatively accurate and is recommended to use for predictive tasks [31]. PFI=1 implies the treatment outcome is unfavorable. For example, the patient had a new tumor event in a fixed period, such as a progression of disease, local recurrence, distant metastasis, new primary tumors, or died with cancer without a new tumor event. PFI=0 means the patient did not have a new tumor event or was censored in a fixed period. We are trying to predict the Progression-Free Interval (PFI) event using four types of -omics data (i.e., gene expression, miRNA expression, protein expression, and DNA methylation). As this is a binary classification problem, we used Average Precision and AUC (Area Under the ROC Curve) score as the main metrics to evaluate classification performances. The results using other metrics are similar.

#### Data preprocessing

We performed log transformation and removed outliers for gene features. Four thousand nine hundred forty two gene features were kept for downstream analysis after filtering out genes with either low mean or low variance. We removed features with low mean and variance for DNA methylation data. Four thousand seven hundred fifty three methylation features (i.e., beta values associated with CpG islands) were selected for analysis. We also performed log transformation and removed outliers for miRNA features. We removed nine protein expression features with NA values. In total, 10,546 features were selected for downstream analysis. For each type of features, we normalize it to have zero mean and standard deviation equal to 1.

##### Molecular interaction networks

We downloaded the protein-protein interaction network from the STRING (v10.5) database [26] (https://string-db.org/), which contains more than ten million protein-protein interactions with confidence scores between 0 and 1000. We filtered out most interaction edges with low confidence scores and selected about 1.5 million interaction edges with confidence scores at least 400. We extracted a subnetwork from this PPI interaction network for gene and protein expression features. Since the gene-gene interaction network is too sparse, we performed a one-step random walk (i.e., multiplying the interaction network by itself), removed outliers and normalized it. For miRNA and methylation features, we first map to miRNA/methylation to gene (protein) features and then calculate a miRNA-miRNA and a methylation-methylation interaction network. Take miRNA data as an example. Let **M**_*miR*−*pro*_ be the adjacency matrix for the miRNA-protein mapping (this matrix is derived from miRDB (http://www.mirdb.org) miRNA target prediction scores), and **M**_*pro*−*pro*_ be the protein-protein interaction network, then the miRNA-miRNA interaction network **M**_*miR*−*miR*_ is calculated as follows:

$$\mathbf{M}_{miR-miR} = \mathbf{M}_{miR-pro} \cdot \mathbf{M}_{pro-pro} \cdot \mathbf{M}_{miR-pro}^{T} $$

All the four feature interaction matrices are normalized to have a Frobenius norm equal to 1.

We randomly chose 70% of the dataset as the training set, 10% as the validation set, and the rest 20% as the test set. We trained different models on the training set and evaluated them on the validation set. We chose the model with the best validation accuracy to make predictions on the test set and reported the Average Precision and AUC score on the test set.

### Experimental results

We compare our model with SVM, Decision Tree, Naive Bayes, Random Forest, and AdaBoost, as well as Variational AutoEncoder (VAE) and Adversarial AutoEncoder (AAE). Traditional models such as SVM only accept one feature matrix as input. So we used the concatenated feature matrix as model input. We used a linear kernel for SVM. We used 10 estimators in Random Forest and 50 estimators in AdaBoost.

For the Multi-view AutoEncoder (MAE) model with a classification head, we used a three-layer neural network. The input layer has 10,546 units (features). Both the first and second hidden layers have 100 hidden units. The last layer also has 10,546 units (i.e., the reconstruction of the input). We added a classification head which is a linear layer with two hidden units corresponding to two classes.

To facilitate fair comparisons, all of our proposed Multi-view Factorization AutoEncoder (MAE) models share the same model architecture(i.e., two hidden layers each with 100 hidden units for each of the four submodule autoencoders), but the training objectives are different. Since this dataset has four different data types, our model has four autoencoders as submodules, each of which encodes one type of data (one view). Figure 1 shows our model structure (note in our experiments we have four views instead of only two shown in the figure). We combine the outputs of the four autoencoders (i.e., the outputs of the last hidden layers) by adding them together (Eq. 12) for classification tasks.

The training objective for the Multiview Factorization AutoEncoder (MAE without graph constraints) includes only the first two terms in Eq. 16. The objective for the Multiview Factorization AutoEncoder with feature interaction network constraints (MAE + feat_int) includes the first three terms in Eq. 16. The objective for the Multiview Factorization AutoEncoder with patient view similarity network constraints (MAE + view_sim) includes the first two and the last terms in Eq. 16. And the objective for the Multiview Factorization AutoEncoder with both feature interaction and view similarity network (MAE + feat_int + view_sim) constraints includes all four terms in Eq. 16.

As our proposed model with network constraints is end-to-end differentiable, we trained it with Adam [32] with weight decay 10^−4^. The initial learning rate is 5×10^−4^ for the first 500 iterations and then decreased by a factor of 10 (i.e., 5×10^−5^) for another 500 iterations. Models with the best validation accuracies are used for prediction on the test set.

The Average Precision and AUC scores for Bladder Urothelial Carcinoma (BLCA) and Brain Lower Grade Glioma (LGG) using these models are shown in Tables 1 and 2. Our proposed models (in bold font) achieved better Average Precision and AUC scores for predicting PFI on both datasets. Note that traditional methods such as Decision Tree do not perform as well as deep learning models. This may be due to the superior representation power of deep learning. The more recent Bayesian deep learning approach Variational AutoEncoder (VAE) did not achieve good results, while Adversarial AutoEncoder (AAE) achieved better results than other methods except our proposed method. Currently, the datasets contain a lot of noise (due to the nature of) and the feature interaction networks derived from public knowledgebases are incomplete and noisy, too. If a larger dataset consisting of hundreds of thousands of patients is available, we expect our proposed model with more network constraints to be able to generalize even better.

**Table 1:** Results for bladder urothelial carcinoma (BLCA) dataset

Model name	Average precision	AUC
SVM	0.587	0.688
Decision tree	0.590	0.575
Naive Bayes	0.456	0.635
Random forest	0.575	0.670
AdaBoost	0.587	0.662
Variational AE	0.528	0.563
Adversarial AE	0.617	0.693
Multi-view AE	0.595	0.699
MAE + feat_int	0.650	0.719
MAE + view_sim	0.652	0.723
**MAE + feat_int + view_sim**	**0.664**	**0.740**

**Table 2:** Results for brain lower grade glioma (LGG) dataset

Model name	Average Precision	AUC
SVM	0.591	0.713
Decision tree	0.518	0.658
Naive Bayes	0.568	0.742
Random forest	0.661	0.670
AdaBoost	0.594	0.673
Variational AE	0.628	0.642
Adversarial AE	0.659	0.702
Multi-view AE	0.551	0.726
MAE + feat_int	0.576	0.727
MAE + view_sim	0.737	0.819
**MAE + feat_int + view_sim**	**0.746**	**0.825**

**Multi-omics outperforms single -omics**We trained our autoencoder models on each type of -omics data (i.e., gene expression, miRNA expression, protein expression, and DNA methylation), and compared them with those trained using multi-omics data (all the four types combined) using our proposed Multi-view Factorization AutoEncoder model. The results on BLCA and LGG datasets are shown in Tables 3 and 4, respectively. For both datasets, the results using multi-omics data (all four data types combined) significantly outperform those using a single type of -omics data.

**Table 3:** Results using single -omics versus multi-omics on BLCA dataset

Model name	Average precision	AUC
Gene (single -omics)	0.532	0.688
miRNA (single -omics)	0.368	0.507
Protein (single -omics)	0.399	0.567
DNA Methylation (single -omics)	0.601	0.634
**Combined multi-omics**	**0.664**	**0.740**

**Table 4:** Results using single -omics versus multi-omics on LGG dataset

Model name	Average precision	AUC
Gene (single -omics)	0.634	0.728
miRNA (single -omics)	0.501	0.686
Protein (single -omics)	0.575	0.735
DNA Methylation (single -omics)	0.610	0.698
**Combined multi-omics**	**0.746**	**0.825**

### Results on the tCGA pan-cancer dataset

We also performed experiments on the TCGA Pan-cancer dataset [1] consisting of 6179 patients with 21 different cancer types. In addition to predicting Progression-Free Interval (PFI) event, we also predict Overall Survival (OS) event. Similar to PFI, OS is another derived clinical (binary) outcome endpoints [31]. OS=1 means for patients who were dead from any cause based on the follow-up data; OS=0 for otherwise. There are 4460 patients with OS=0 and 1719 patients with OS=1. PFI and OS are the same for most cases (4941 out of 6179, or 80%). There are 4268 patients with PFI=0 and 1911 patients with PFI=1. PFI is preferred over OS given the relatively short follow-up time. Therefore, we mainly use PFI as a binary target.

We used the same data processing procedure and experimental settings as described above for the BLCA and LGG datasets. The average AUC scores (10 runs) for predicting PFI and OS using these models are shown in Table 5. Our proposed models (in bold font) achieved better AUC scores for both predicting PFI and OS than other traditional machine learning methods.

**Table 5:** AUC scores for predicting PFI and OS on the TCGA pan-cancer dataset

Model name	AUC (OS)	AUC (PFI)
SVM	0.699	0.625
Decision Tree	0.670	0.634
Naive Bayes	0.655	0.644
kNN	0.706	0.659
Random Forest	0.720	0.661
AdaBoost	0.716	0.689
MAE + feat_int	0.765	0.721
**MAE + view_sim**	0.763	**0.724**
**MAE + feat_int + view_sim**	0.766	**0.724**

In order to study if the model architecture would significantly affect the results, we change the number of hidden layers from one layer to three layers. The number of units for each hidden layer is shown in Table 6. (Note we have omitted the input and output layers both of which have 10,546 hidden units). As shown in Table 6, the results are not significantly different. In addition, we had tried to use DenseNet [30] and ResNet [29] as the backbone of the autoencoders instead of multi-layer perceptrons. The results are also not significantly different and thus not presented here.

**Table 6:** AUC scores for PFI with different model architectures

Number of hidden units	AUC (PFI)
100	0.723
200	0.725
200-100	0.726
100-200	0.727
100-100	0.724
100-100-100	0.725
50-100-200	0.726
100-50-200	0.726
200-100-50	0.722

#### Learned feature embeddings preserve interaction network structure

**Fig. 2:**
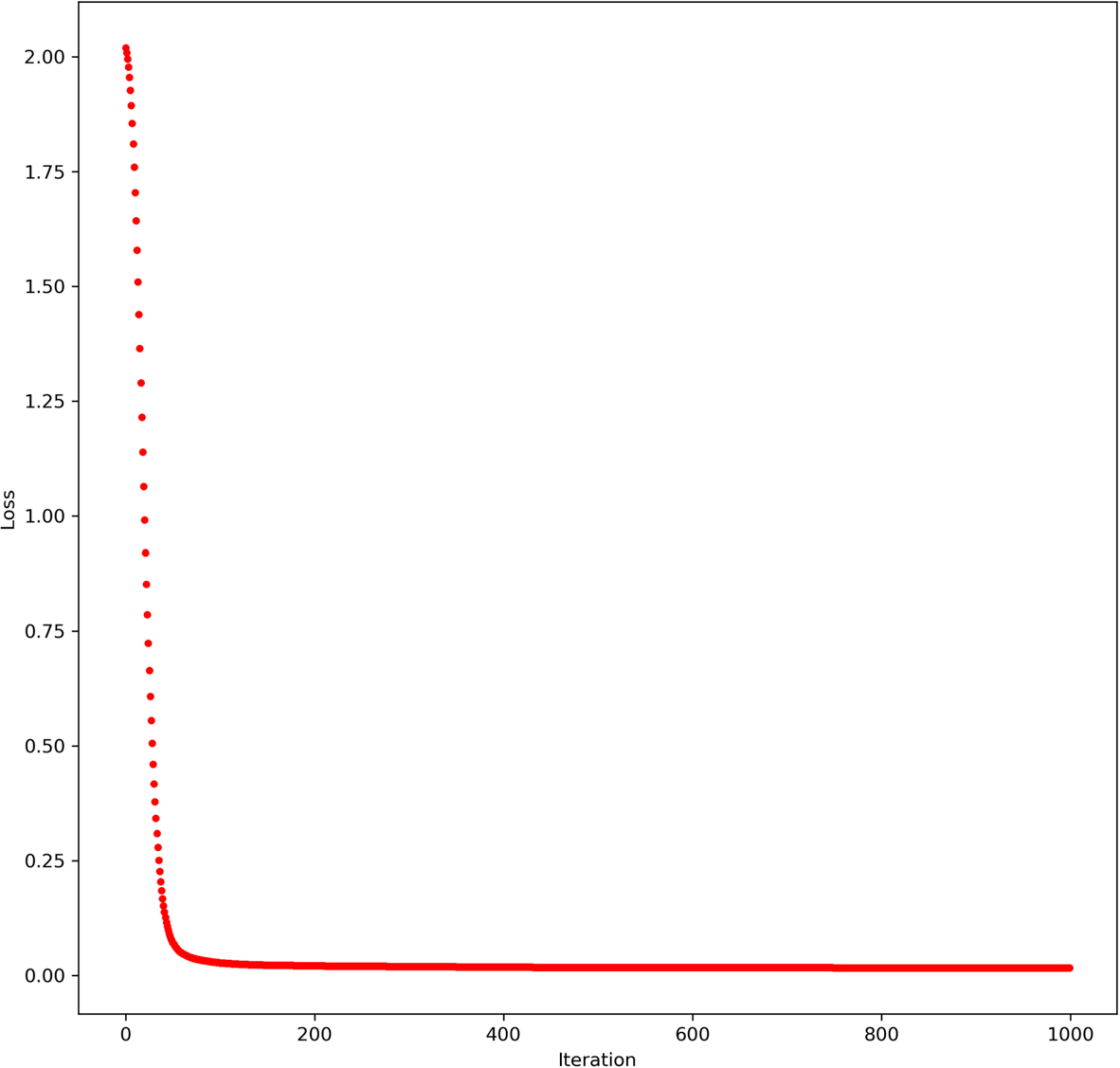
A typical feature interaction network regularizer training loss curve. After about 100 iterations, the training loss (corresponding to the third term in Eq. 16) approaches almost zero, which means the learned molecular feature embeddings become consistent with the provided feature interaction networks

Our proposed model learns patient representations and feature embeddings simultaneously. While patients are different from datasets to datasets, the genomic features (such as gene features) and their interaction networks are from domain knowledge, and thus are persistent regardless of which dataset we are using. Since we have a regularization term in the loss to ensure the learned feature embeddings are consistent with feature interaction networks, we would like to know if the model is able to learn an embedding that is “compatible” with the domain knowledge of interaction networks. We plotted the loss term $\sum _{v=1}^{V} Trace\left (\mathbf {Y}^{(v)} \cdot \mathbf {L}_{G^{(v)}} \cdot \mathbf {Y}^{(v)^{T}}\right)$ from one typical run of training our model with feature interaction network constraints in Fig. 2. This regularization term decreased to nearly zero very fast, which means the information from feature interaction networks is fully assimilated into the model, or more specifically, the weights of the decoders in the model. We found that many independent runs show very similar loss curves, which means the model is able to robustly learn a feature embedding that preserves the feature interaction network information.

## Conclusion

While it is challenging for applying machine learning to multi-omics data with the “big p, small n” problem, biological domain knowledge can be incorporated into the machine learning model as inductive biases to alleviate potential overfitting problems. A number of knowledgebases (e.g., STRING [26], Reactome Pathways [27], etc.) contain the information for extracting biological interaction networks, which can be incorporated into various machine learning models. In this paper, we presented the Multi-view Factorization AutoEncoder (MAE) model with network constraints that can effectively integrate domain knowledge such as molecular interaction networks with multi-omics data for accurately predicting clinical outcomes.

The MAE model consists of multiple factorization autoencoders as submodules for individual data types (views) and combines multiple views with their high-level abstract representations for supervised learning. The factorization autoencoder employs a deep architecture for the encoder and a shallow architecture for the decoder. This increases the overall model representation power and provides a natural way to integrate graph constraints into the model. Our model learns molecular and patient embeddings simultaneously. With effective network regularization techniques, we can learn good feature representations and consistent patient similarity networks and feature interaction networks.

The experimental results on the Bladder Urothelial Carcinoma (BLCA) and Brain Lower Grade Glioma (LGG) datasets and the TCGA pan-cancer dataset demonstrated that our proposed model with feature interaction network and patient similarity network constraints outperforms traditional methods and conventional deep learning models on predicting clinical target variables from multi-omics data. Our method can be applied to other large-scale multi-omics datasets to learn latent representations that are consistent with molecular interaction networks for various molecular entities. Besides multi-omics data, our proposed method can also be applied to any other multi-view data with feature interaction networks.

The ultimate goal of multi-omics data integrative analysis is to disentangle complex factors and identify important factors that contribute to disease etiology. Our model learns distributed representations for various molecular entities and facilitates mining relationships among molecular features and clinical features. Essentially, learning good representations for both molecular and clinical features is fundamentally important to unravel the intricate relationships among them. Our work also provides a proof-of-concept framework for unifying data-driven and knowledge-driven approaches for mining multi-omics data with biological knowledge. We hope it can be applied to large-scale cancer genomics data and contribute to elucidating the etiology and mechanisms of cancer and other complex genetic diseases.

## Data Availability

All the data used in the experiments are publicly available at the GDC Data Portal website (https://portal.gdc.cancer.gov/).

## Abbreviations

AAE: Adversarial AutoEncoder
ANF: Affinity network fusion
AUC: Area under the ROC curve
BLCA: Bladder urothelial carcinoma
LGG: Brain lower grade glioma
MAE: Multi-view factorization AutoEncoder
OS: Overall survival
PFI: Progression-free interval
SNF: Similarity network fusion
TCGA: The cancer genome atlas
VAE: Variational AutoEncoder
